# Value-Based Pricing and Budget Impact Analysis for Multi-Indication Drugs: A Case Study of Immunotherapies

**DOI:** 10.3390/ijerph19074105

**Published:** 2022-03-30

**Authors:** So-Young Ha, Dong-Won Kang, Hye-In Jung, Eui-Kyung Lee, Mi-Hai Park

**Affiliations:** School of Pharmacy, Sungkyunkwan University, Suwon 16419, Korea; patty21c@gmail.com (S.-Y.H.); first123east@gmail.com (D.-W.K.); jasmine9614@gmail.com (H.-I.J.)

**Keywords:** value-based pricing, multi-indication, immunotherapy, indication-based pricing, weighted-average pricing

## Abstract

We aimed to calculate the value-based price of each indication and compare the drug price and budget impact among value-based pricing (VBP) scenarios, using immunotherapy as a case. Atezolizumab, nivolumab, and pembrolizumab prices were estimated for VBP scenarios, namely indication value-based pricing (IBP), IBP with refund, and weighted-average pricing (WAP). To estimate the value-based price of each indication, cost-effectiveness analyses were conducted by setting the incremental cost-effectiveness ratio of the first reimbursed indication to the threshold. The budget impact for each scenario was compared with that of the pricing system in Korea (which has a 4.75% price reduction). The value-based prices of non-reimbursed indications were lower for atezolizumab and higher for nivolumab than those for the reimbursed indication. The drug price fluctuations were the largest in IBP, varying between 28.56–328.81% of the current list price. The net price of the non-reimbursed indications decreased from 0% to 71.44% in IBP with refund, and the budget impact was the lowest among VBPs. Although the fluctuation in the budget impact in WAP was smaller than IBP, higher drug prices were identified for low-value indications. In conclusion, IBP with refund is a viable method for multi-indication drugs, because it has minimal drug price and budget impact changes.

## 1. Introduction

Globally, 57 anticancer drugs have been approved for 89 indications between 2014 and 2018 [1]. In particular, immunotherapy, a treatment that activates the immune system, has been approved for a wide range of indications owing to its ability to treat various types of cancer [2]. As the number of multi-indication drugs is on the rise, 78% of the stakeholders surveyed, including industry, regulators, payers, and academicians, have agreed to the need for a pricing system that reflects the value of each indication [3]. In addition, since the increased usage of costly drugs due to the expansion of approved indications imposes a financial burden on the payers [4], the decision for reimbursement should consider the level of the budget impact for each indication. However, if drug prices are lowered without considering the clinical value of each indication upon the addition of subsequent indications, it may hinder the development of pharmaceutical manufacturers to expand their scope for different indications [5]. Therefore, value-based pricing (VBP), which reflects the value of each indication while minimizing the burden on the payer, is required to increase the accessibility of multi-indication drugs.

VBP evaluates the value, defined as the clinical benefit of treatment with respect to its cost [6], and then applies it to the drug price [7]. Various VBP methods can be used to determine the price of multi-indication drugs. First, the indication value-based price approach applies different prices to each indication [8]. Second, the hybrid approach maintains a single list price but varies the net price by applying different refund rates or different risk-sharing assessment (RSA) schemes for each indication. Third, the weighted average approach calculates a single price using the weighted average of the value-based price of each indication by the expected amount of usage [9]. Italy, Belgium, France, and Switzerland vary their net prices for each indication using the hybrid approach [10]. In Germany, drug price is renegotiated by considering the weighted average value-based price for each indication whenever one is added [11]. In Korea, the value of each indication is not considered upon the addition of subsequent indications, and the drug price is lowered considering the budget impact, except for some indications with a small budget impact [5].

Although previous studies suggest various VBPs, only a few of these quantitatively compare the prices of multi-indication drugs among VBPs. Bach et al. calculated prices for each indication based on a threshold of $150,000 per year of life gain [6]. Yeung et al. calculated the indication value-based prices using clinical trials and real-world data and compared them with the current single price [12]. O’Donnell et al. estimated the drug prices for several VBP scenarios [9]. However, previous studies have simply estimated drug prices using mean or median survival data, rather than modeling cost-effectiveness analysis for patients. In addition, no studies have compared the budget impact on VBPs, although the budget impact is an important consideration in determining reimbursement and pricing within limited resources [4].

In this study, we aimed to compare various VBP methods and present the reasonable pricing method with the smallest budget impact for multi-indication drugs using immunotherapy as a case study. We measured the value-based price for each indication by performing a cost-effectiveness analysis and comparing drug prices among VBPs. In addition, we compared the budget impact for each VBP scenario with the pricing system in Korea, which does not reflect the value of the expanded indication.

## 2. Materials and Methods

This study was conducted in three steps. The first step was to select multi-indication immunotherapies and identify approved and reimbursed indications for these drugs. In the second step, we calculated the value-based price for each non-reimbursed indication of immunotherapy using cost-effectiveness analysis and compared the drug price among the following three VBP scenarios: indication value-based pricing (IBP), IBP with a refund, and weighted average pricing (WAP). In the last step, the budget impact was assessed for the three VBP scenarios and compared with the pricing system in Korea (which has a 4.75% price reduction).

### 2.1. Selection of the Multi-Indication Immunotherapies

We selected reimbursed multi-indication immunotherapies with two or more indications approved by the Ministry of Food and Drug Safety up to September 2020. Clinical trials for the approved indications of selected drugs were confirmed using ClinicalTrials.gov by the U.S. National Library of Medicine [13]. Reimbursed or non-reimbursed indications were identified through the Health Insurance Review and Assessment Service (HIRA) in Korea [14]. For the approved multi-indication immunotherapies, information on the reimbursement status of the indications, intervention, and comparator of the randomized clinical trials was collected.

### 2.2. Cost-Effectiveness Analysis and Scenarios for VBP

We conducted a cost-effectiveness analysis to measure the value-based price for each indication of the selected drug. Clinical effectiveness was identified as the survival outcomes of each indication, including the progression-free survival (PFS) and overall survival (OS), from phase 3 clinical trials. To estimate the long-term survival outcomes, we obtained the whole survival information by extracting the data from published Kaplan–Meier survival curves for both the treatment and the control groups using algorithms suggested in a previous study [15]. The Enguage Digitizer and “ipdfc” packages provided by STATA were used to reconstruct the data [16]. In the process of extrapolation based on the reconstructed patient-level data, the proportional hazard assumptions from the OS and PFS curves were confirmed using the log cumulative hazard plots and Schoenfeld residual plots. We estimated the distribution using the dependent model when the assumptions were satisfied; otherwise, we used the independent model. Among the “Exponential,” “Weibull,” “Gompertz,” “Log-normal,” “Log-logistic,” and “Generalized gamma” distributions, the best fit was selected based on the Akaike Information Criterion, Bayes Information Criterion, and visual plotting. The “flexsurv” package in R was used for survival analysis. The selected distributions and parameters of OS and PFS curves are presented in Supplementary Table S1.

A partitioned survival model with the health state of PFS and progressive disease (PD) was used to assess cost-effectiveness. The time horizon was set to seven years, considering the average period in which the survival rate of patients identified by survival analysis was 10% or less. The cycle length was set to one month. Quality-adjusted life-years (QALYs) were estimated by applying the utility from a systematic review of PFS or progression to life years. Using the micro-costing approach, the costs for treatment, dispensing, outpatient visits, and monitoring were calculated. The costs for treatment refer to the drug cost including immunotherapy or subsequent therapy, and the dispensing fee is the cost for administering these drugs. The costs for outpatient visits include the consultation fee of doctors, and the costs for monitoring include the routine testing and the special testing for each indication that is necessary to administer drugs. Adverse events and end-of-life costs were calculated using the macro-costing approach [17]. Among the grade 3 or higher adverse events reported in the clinical trial for each indication, the medical cost for only those with an incidence of at least 5% was applied in this model. The input data used for cost-effectiveness analysis are presented in Supplementary Table S2.

The incremental cost-effectiveness ratio (ICER) was derived by dividing the incremental QALYs by the incremental costs for each indication based on the list of drug prices of the first reimbursed indication from the HIRA. We set the ICER of the first reimbursed indication to the threshold and calculated the value-based price of each indication that met the threshold for each drug. We de-identified the ICERs and calculated them for each indication in multiples based on the first reimbursed indication. This was done because the price of immunotherapies does not correspond to the net price due to RSA schemes, such as refunds, and the main purpose of this study was to compare the value-based price of each indication, and not the ICER.

We developed three scenarios for VBP based on previous studies [8,9,10] and calculated the drug price in each scenario. In the first scenario (Scenario 1: IBP), we applied the drug price differently for each indication, depending on the value-based price. In the second scenario (Scenario 2: IBP with a refund), we varied the net price for each indication through the refund rate while maintaining a single current list price. In this scenario, the ratio of list price to net price was the refund rate. When the price was higher than the current list price, the net price was set as the list price with no refunds. The third scenario (Scenario 3: WAP) employed weighted averaging for the value-based price of each indication against the expected amount of their usage.

### 2.3. Budget Impact Analysis

We performed a three-year budget impact analysis from the payer’s perspective to identify the financial impact of each non-reimbursed indication for each of the three scenarios. The budget impact was calculated using the expected number of patients, expected market share, and annual treatment costs. The expected number of patients was projected using the past five-year prevalence of cancer between 2013 and 2017 and the annual increase rate of cancer in South Korea [18]. Annual treatment costs were calculated by adding the administration costs and the annual drug costs. The market share was assumed to be 30% in the first year, 40% in the second year, and 50% in the third year, reflecting the compound annual growth rate (CAGR) of 10% of immunotherapies. The input data used for the budget impact analysis are presented in Supplementary Table S3. We compared the budget impacts for each VBP with the pricing system in Korea, which applied the average drug price reduction rate (4.75%) with the expansion of reimbursed indications [19], not just to compare among the VBP scenarios, but also to identify the differences between each VBP scenario and the pricing system that did not reflect the value for the expanded indication.

## 3. Results

Among the immunotherapies, atezolizumab, nivolumab, and pembrolizumab were selected for our analysis (Supplementary Table S4). The second-line non-small cell lung cancer (NSCLC) was the only reimbursed indication for atezolizumab, nivolumab, and pembrolizumab as of September 2020. The number of non-reimbursed indications was the highest for pembrolizumab.

The ICERs of atezolizumab in the two non-reimbursed indications were 2.8 and 2.0 times higher than those in the reimbursed indications. Although the incremental QALYs of atezolizumab were the same in the reimbursed indications and first-line metastatic NSCLC, the ICER was 2.8 times higher in the first-line metastatic NSCLC, owing to its higher cost by 2.9 times. The ICERs of nivolumab in second-line advanced renal cell carcinoma (RCC) and the second-line recurrent or metastatic head and neck squamous cell carcinoma (HNSCC) were 0.7 and 0.2 times higher relative to the first reimbursed indication, respectively, due to the higher incremental QALYs and lower incremental costs. The ICERs of pembrolizumab varied between 0.5–2.4 times relative to the reimbursed indications (Table 1).

As a result of the cost-effectiveness of each indication, setting the ICER of the first reimbursed indication as the threshold, the price in scenario 1 (IBP) was lower than that of the first reimbursed indication for atezolizumab. Because of the lower price than the list price, the refund rates in scenario 2 (IBP with refund) were 71.44% for first-line metastatic NSCLC and 58.58% for first-line locally advanced or metastatic TNBC. In nivolumab, the price of non-reimbursed indications in scenario 1 (IBP) was about 1.4 to 3.3 times higher than that of the first reimbursed indication, with the refund rates as 0% in scenario 2 (IBP with refund). Lastly, the prices in scenario 1 (IBP) varied from 59.55% to 169.45% of the price of the first reimbursed indication for pembrolizumab. The WAPs for atezolizumab, nivolumab, and pembrolizumab in Scenario 3 were 68.24%, 123.76%, and 96.61% of the current list price, respectively (Table 2).

Compared to the pricing system in Korea (which has a 4.75% price reduction), the budget impact of atezolizumab (−49.42%) and pembrolizumab (−11.79%) decreased, whereas that of nivolumab (475.51%) increased in scenario 1 (IBP). The budget impact in scenario 2 (IBP with refund) was the lowest among the other scenarios, as it decreased with atezolizumab (−49.42%) and pembrolizumab (−19.34%), and increased with nivolumab (13.27%). For scenario 3 (WAP), the budget impact of atezolizumab (−20.30%) decreased, while that of nivolumab (79.65%) and pembrolizumab (15.05%) increased (Table 3).

**Figure 1:**
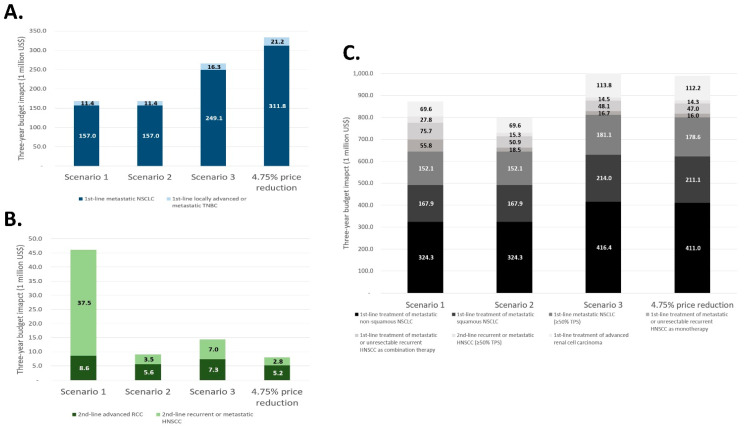
The budget impact for each non-reimbursed indication: (**A**) atezolizumab, (**B**) nivolumab, and (**C**) pembrolizumab.

In atezolizumab, the budget impact of first-line metastatic NSCLC and first-line locally advanced or metastatic TNBC in scenario 1 (IBP) and scenario 2 (IBP with refund) was approximately half that of the pricing system in Korea (which has a 4.75% price reduction). In nivolumab, the budget impact of second-line recurrent or metastatic HNSCC was about 10.7 times higher in scenario 1 (IBP) compared to scenario 2 (IBP with refund). In pembrolizumab, the budget impact decreased in the first-line treatment of metastatic NSCLC and RCC in scenarios 1 (IBP) and 2 (IBP with refund), but slightly increased in scenario 3 (WAP) compared to the pricing system in Korea (which has a 4.75% price reduction) (Figure 1).

## 4. Discussion

The findings of our study empirically demonstrate that the drug price and budget impacts change according to the value of expanded indication in each VBP scenario. Among the three VBP scenarios, IBP, which reflected its value in the drug price, could be an alternative approach for the country in adopting health technology assessment (HTA). Cole et al., who performed a survey on decision-makers in 16 European countries, report that accessibility to new drugs can increase with IBP [7]. However, this method has a shortcoming in that fluctuations in drug prices and budget impacts can be very large, depending on the value of the individual indications. In this study, the drug price of nivolumab in second-line recurrent or metastatic HNSCC increased by approximately 3.5 times its current list price, and the budget impact increased by 5 to 13 times to the cost of other scenarios. However, the price of atezolizumab in first-line metastatic NSCLC decreased to 28.6% of the current list price, and the budget impact was half that of the pricing system in Korea. The price of pembrolizumab with numerous approved indications was approximately 60–169% of the current list price. These fluctuations may cause inequality among patients with high-value indications because of higher prices as compared to those with low-value indications, [20] and they may also sharply increase the financial burden, as was the case with nivolumab. Therefore, a system that can accommodate and manage these fluctuations is required for IBP.

IBP with refund showed the least budget impact for all immunotherapies among the VBPs due to the decrease in the net price for low-value indications and the reduction in the risk of budget increase for high-value indications. The net price for the non-reimbursed indication decreased from 0% to 71.44%, while maintaining the current list price. In atezolizumab and pembrolizumab, the lower net price in low-value indications reduced the budget impact in IBP with refund. In nivolumab, with a higher value for non-reimbursed indication as compared to the first reimbursed indication, the current list price acts as an upper limit, thereby suppressing the risk of the budget increase. This approach can also minimize confusion about fluctuations in drug prices by maintaining a single list price. Given these benefits, IBP with a refund is considered the most reasonable alternative to the pricing method for multiple indications. However, there is a limitation in that the value of indications, which is higher than that of the first reimbursement, was not reflected in the drug price in IBP with refund. In particular, it was difficult to reflect the value of the later-expanded indications in the drug price if the value of the first reimbursed indication was very low.

WAP has already been adopted in several countries, owing to its ease of access [21]. In WAP, the fluctuation in budget impacts according to the value of the indication are smaller than those of the IBP. However, the weighted averaging may result in high drug prices, even for low-value indications. The WAP of atezolizumab, with a lower value for the expanded indication than that for the first reimbursed indication, was approximately 32% lower than the current list price. However, this price was much higher than that of the low-value non-reimbursed indication, and the expected budget impact was approximately 30% higher than that of the IBP approach, owing to the large number of patients in each indication.

We presented the pricing system in Korea and compared it among different scenarios. In Korea, adopting HTA, while the price of the first reimbursed indication is determined by evaluating the value, most drug prices are lowered (average of 4.75%) without considering their value when the reimbursed indications are expanded. Because this approach does not correctly reflect the value in the drug price, it can increase the budget impact for low-value indications as compared to VBP. Our findings show that the budget impact of atezolizumab decreased in all VBP scenarios as compared to the current pricing system in Korea. A pricing system that does not reflect value can also affect the development of pharmaceutical manufacturers to expand their indications. Pharmaceutical manufacturers may delay the expansion of reimbursed indications because of concerns about reduced drug prices. A previous study that surveyed pharmaceutical employees in Korea reported that a drug pricing system that does not consider value reduces accessibility to multi-indication drugs [5]. Therefore, if the drug price is determined without considering the value of each indication, it not only decreases the accessibility of multi-indication drugs but also increases the budget impact for drugs with low-value indications.

The value-based price of each indication in our study was 0.28 to 3.3 times compared to the price of the reimbursed indication. Similarly, previous studies report various prices for each indication. Bach et al. showed that the price of nab-paclitaxel, erlotinib, cetuximab, and trastuzumab for each indication was approximately 2–22 times higher than the prices of each drug for the lowest-value indication [6]. Yeung et al. showed that the price of trastuzumab was approximately 3.76 times higher for metastatic breast cancer than for advanced gastric cancer [12]. However, the long-term survival outcomes are not reflected in the drug price because these previous studies used only the median or mean survival gain from clinical trials to estimate effectiveness. Our study is meaningful in that it is the first to estimate the value-based price for each indication by performing a cost-effectiveness analysis by reconstructing survival data and estimating long-term effects. In addition, we not only quantitatively calculated the drug price between various VBP scenarios, but also analyzed the budget impacts based on these calculated prices.

This study has some limitations. First, the estimated survival data obtained by reconstructing the digitizing algorithms may differ from the actual clinical effects of the drug. However, this algorithm is most often used to construct survival data, and a previous study reports the accuracy of this method [15]. Second, since we could not identify the actual negotiated price of the selected immunotherapies due to the confidential RSA contract, the results of the calculated price and budget impacts may be unreliable. However, as the purpose of this study was to compare the drug prices and budget impacts among VBP scenarios, the actual drug price does not affect the results. Third, as there was no data available to confirm the market share of immunotherapy by indications, the market shares in the first, second, and third years of the expansion of reimbursement criteria for budget impact analysis were assumed. Moreover, calculating the number of patients corresponding to the approved indications was difficult, as it was necessary to assume the gene expression and failure rate of previous treatment in the verifiable data. Finally, as the comparator in the cost-effectiveness analysis was selected by referring to the phase 3 clinical trial for each indication, the results may vary depending on the selection criteria for the comparator.

## 5. Conclusions

In conclusion, IBP with refund is considered the most reasonable alternative to the pricing method for multiple indications with the lowest budget impact due to a decrease in the net price for low-value indication while reducing the risk of budget increase for high-value indications with a ceiling price. The fluctuations in the drug price and budget impacts in IBP were the largest, depending on the value of the individual indications. In WAP, although the fluctuation in budget impact was smaller than that of IBP, it may result in higher drug prices even for low-value indications, thereby causing a substantial financial burden. This first study estimating the value-based price through cost-effectiveness analysis with the long-term survival outcomes suggests a way to more precisely reflect the value in the price of multi-indication drugs, and this method can be applied to compare various VBP systems quantitatively. Our findings are expected to provide evidence for determining the pricing system for multi-indication drugs, including immunotherapies.

## Figures and Tables

**Table 1:** The results for cost-effectiveness analysis for each indication of immunotherapy.

Indication	Treatment	QALY		Cost (USD)		ICER (USD/QALY)
		Expected Value	Difference	Expected Value	Difference	
Atezolizumab						
Second-line NSCLC (reimbursed indication)	Intervention	1.23	0.29	a	c	d
	Comparator	0.84		b		
First-line metastatic NSCLC	Intervention	1.36	0.29	2.3a	2.9c	2.8d
	Comparator	1.07		2b		
First-line locally advanced or metastatic TNBC	Intervention	1.39	0.46	2a	3.3c	2.0d
	Comparator	0.93		1.5b		
Nivolumab						
Second-line NSCLC (reimbursed indication)	Intervention	1.09	0.37	e	g	h
	Comparator	0.72		f		
Second-line advanced RCC	Intervention	2.40	0.44	1.8e	0.8g	0.7h
	Comparator	1.96		3.6f		
Second-line recurrent or metastatic HNSCC	Intervention	0.93	0.45	0.5e	0.2g	0.2h
	Comparator	0.48		0.9f		
Pembrolizumab						
Second-line NSCLC (reimbursed indication)	Intervention	1.60	0.80	i	k	l
	Comparator	0.80		j		
First-line treatment of metastatic non-squamous NSCLC	Intervention	1.59	0.42	1.0i	0.8k	1.4l
	Comparator	1.17		2.3j		
First-line treatment of metastatic squamous NSCLC	Intervention	1.45	0.51	0.8i	0.8k	1.3l
	Comparator	0.94		0.9j		
First-line metastatic NSCLC (≥50% TPS)	Intervention	2.36	1.09	1.5i	1.7k	1.2l
	Comparator	1.28		0.6j		
First-line treatment of metastatic or unresectable recurrent HNSCC as monotherapy	Intervention	1.38	0.53	0.6i	0.3k	0.5l
	Comparator	0.84		2.1j		
First-line treatment of metastatic or unresectable recurrent HNSCC as combination therapy	Intervention	1.19	0.36	0.5i	0.3k	0.7l
	Comparator	0.84		2.1j		
Second-line recurrent or metastatic HNSCC (≥50% TPS)	Intervention	1.07	0.53	0.5i	0.4k	0.6l
	Comparator	0.54		1.1j		
First-line treatment of advanced renal cell carcinoma	Intervention	3.29	0.32	1.8i	1.0k	2.4l
	Comparator	2.97		7.1j		

Abbreviations: QALY, quality-adjusted life year; ICER, incremental cost-effectiveness ratio; NSCLC, non-small cell lung-cancer; TNBC, triple-negative breast cancer; RCC, renal cell carcinoma; HNSCC, head and neck squamous cell carcinoma; TPS, tumor proportion score; USD, United States Dollar. a, e, i: the expected cost of the intervention group in reimbursed indication; b, f, j: the expected cost of the comparator group in reimbursed indication; c, g, k: difference in expected cost between the intervention group and comparator group in reimbursed indication, d, h, l: the incremental cost-effectiveness ratio in reimbursed indication.

**Table 2:** Drug price of immunotherapies according to each value-based pricing scenario.

Indication	Current List Price (USD)	Value-Based Pricing				4.75% Price Reduction, USD (%)
		Scenario 1: IBP *, USD (%)	Scenario 2: IBP with Refund		Scenario 3: WAP, USD (%)	
			Price (USD)	Refund Rate (%)		
Atezolizumab						
Second-line NSCLC (reimbursed indication)	2049 (reference)	2049 (100.00%)	2049	0.00%	1398 (68.24%)	1952 (95.25%)
First-line metastatic NSCLC		585 (28.56%)		71.44%		
First-line locally advanced or metastatic TNBC		849 (41.42%)		58.58%		
Nivolumab						
Second-line NSCLC (reimbursed indication)	1172 (reference)	1172 (100.00%)	1172	0.00%	1450 (123.76%)	1116 (95.25%)
Second-line advanced RCC		1643 (140.24%)		0.00%		
Second-line recurrent or metastatic HNSCC		3853 (328.81%)		0.00%		
Pembrolizumab						
Second-line NSCLC (reimbursed indication)	2527 (reference)	2527 (100.00%)	2527	0.00%	2441 (96.61%)	2407 (95.25%)
First-line treatment of metastatic non-squamous NSCLC		1861 (73.63%)		26.37%		
First-line treatment of metastatic squamous NSCLC		1896 (75.03%)		24.97%		
First-line metastatic NSCLC (≥50% TPS)		2055 (81.31%)		18.69%		
First-line treatment of metastatic or unresectable recurrent HNSCC as monotherapy		4282 (169.45%)		0.00%		
First-line treatment of metastatic or unresectable recurrent HNSCC as combination therapy		3299 (130.53%)		0.00%		
Second-line recurrent or metastatic HNSCC (≥50% TPS)		4028 (159.38%)		0.00%		
First-line treatment of advanced renal cell carcinoma		1505 (59.55%)		40.45%		

Abbreviations: IBP, indication value-based pricing; WAP, weighted average pricing. * Value-based price is the same as the drug price in indication value-based pricing.

**Table 3:** The budget impact for non-reimbursed indication of immunotherapies according to each value-based pricing scenario.

	Value-Based Pricing			4.75% Price Reduction
	Scenario 1: IBP	Scenario 2: IBP with Refund	Scenario 3: WAP	
Atezolizumab				
Budget impact, USD	168,448,872	168,448,872	265,447,233	333,057,487
Increased rate	−49.42%	−49.42%	−20.30%	-
Nivolumab				
Budget impact, USD	46,082,608	9,069,671	14,384,780	8,007,269
Increased rate	+475.51%	+13.27%	+79.65%	-
		Pembrolizumab		
Budget impact, USD	873,195,849	798,495,489	1,004,647,710	989,960,348
Increased rate	−11.79%	−19.34%	+1.48%	-

## Data Availability

The data presented in this study are available upon reasonable request from the corresponding authors.

## Supplementary Materials

The following supporting information can be downloaded at: https://www.mdpi.com/article/10.3390/ijerph19074105/s1, Supplementary Table S1: The distributions and parameters of overall survival and progression-free survival curves obtained by survival analysis; Supplementary Table S2: Key input data for the cost-effectiveness model; Supplementary Table S3: Key input data for the budget impact analysis; Supplementary Table S4: Comparator and randomized clinical trial for each indication of immunotherapy.
