# Supplementary material for: Value-Based Pricing and Budget Impact Analysis for Multi-Indication Drugs: A Case Study of Immunotherapies
Source: Int J Environ Res Public Health. 2022 Mar 30;19(7):4105. doi: 10.3390/ijerph19074105 (PMC8998444; doi:10.3390/ijerph19074105)
Supplement: Supplementary file 1 [file ijerph-19-04105-s001.zip › ijerph-1630597-SM.pdf]

Supplementary Table S1. The distributions and parameters of overall survival and progression-free survival curves obtained by survival analysis

Indication	Treatment	Overall survival			Progression-free Survival		
		Distribution	Parameter		Distribution	Parameter	
**Atezolizumab**							
2nd-line NSCLC (reimbursed indication)	Intervention	Log-logistic	Shape	1.3617	Gen.gamma	Mu	0.9926
						Sigma	1.1601
			Scale	13.1451		Q	-0.4318
	Comparator	Log-logistic	Shape	1.3617	Log-normal	Meanlog	1.2961
			Scale	13.1451		Sdlog	0.9062
1st-line metastatic NSCLC	Intervention	Gompertz	Shape	0.02282	Log-logistic	Shape	1.6283
			Rate	0.028441		Scale	8.9497
	Comparator	Weibull	Shape	1.2436	Log-logistic	Shape	1.6283
			Scale	20.8296		Scale	8.9497
1st-line locally advanced or metastatic TNBC	Intervention	Log-logistic	Shape	1.613	Log-normal	Meanlog	2.0136
			Scale	7.477		Sdlog	1.0541
	Comparator	Weibull	Shape	1.092	Log-normal	Meanlog	2.0136
			Scale	7.8449		Sdlog	1.0541
**Nivolumab**							
2nd-line NSCLC (reimbursed indication)	Intervention	Log-normal	Meanlog	2.3978	Gen.gamma	Mu	0.9081
						Sigma	1.2775
			Sdlog	1.3665		Q	-0.7629
	Comparator	Log-logistic	Shape	1.61	Log-normal	Meanlog	1.245
			Scale	8.1392		Sdlog	0.9107
2nd-line advanced RCC	Intervention	Log-normal	Meanlog	3.3352	Gen.gamma	Mu	1.1203
						Sigma	0.7785
			Sdlog	1.2084		Q	-1.5512
	Comparator	Log-normal	Meanlog	3.3352	Gen.gamma	Mu	1.3078
						Sigma	0.7913
			Sdlog	1.2084		Q	-0.9403
2nd-line recurrent or metastatic HNSCC	Intervention	Log-normal	Meanlog	2.0056	Log-logistic	Shape	2.022
			Sdlog	1.3701		Scale	2.465
	Comparator	Log-logistic	Shape	1.804	Log-logistic	Shape	2.269
			Scale	5.104		Scale	2.488
**Pembrolizumab**							
2nd-line NSCLC (reimbursed indication)	Intervention	Log-normal	Meanlog	2.8782	Gen.gamma	Mu	1.0281
						Sigma	1.262
			Sdlog	1.5718		Q	-1.508
	Comparator	Log-normal	Meanlog	2.8782	Log-normal	Meanlog	1.3965
			Sdlog	1.5718		Sdlog	1.0339
1st-line treatment of metastatic non-squamous NSCLC	Intervention	Exponential	Rate	0.03135	Log-logistic	Shape	1.4816
						Scale	9.2301
	Comparator	Log-normal	Meanlog	2.5031	Log-logistic	Shape	1.4816
			Sdlog	1.2887		Scale	9.2301

1st-line treatment of metastatic squamous NSCLC	Intervention	Weibull	Shape<br>Scale	1.1752<br>24.2974	Log-logistic	Shape<br>Scale	1.4915<br>8.3228
	Comparator	Weibull	Shape<br>Scale	1.1752<br>24.2974	Log-logistic	Shape<br>Scale	1.4915<br>8.3228
1st-line metastatic NSCLC (≥50% TPS)	Intervention	Gompertz	Shape<br>Rate	-0.03856<br>0.03986	Gompertz	Shape<br>Rate	-0.1052<br>0.1124
	Comparator	Gompertz	Shape<br>Rate	-0.03856<br>0.03986	Log-normal	Meanlog<br>Sdlog	1.5225<br>0.9494
1st-line treatment of metastatic or unresectable recurrent HNSCC as monotherapy	Intervention	Log-normal	Meanlog<br>Sdlog	2.5166<br>1.3798	Gen.gamma	Mu<br>Sigma<br>Q	0.9879<br>1.1172<br>-0.6969
	Comparator	Weibull	Shape<br>Scale	1.2255<br>15.2282	Log-logistic	Shape<br>Scale	1.956<br>4.889
1st-line treatment of metastatic or unresectable recurrent HNSCC as combination therapy	Intervention	Exponential	Rate	0.0507	Log-logistic	Shape<br>Scale	1.5703<br>4.9135
	Comparator	Weibull	Shape<br>Scale	1.2512<br>15.1327	Log-logistic	Shape<br>Scale	2.002<br>4.906
2nd-line recurrent or metastatic HNSCC (≥ 50% TPS)	Intervention	Exponential	Rate	0.05777	Log-logistic	Shape<br>Scale	1.369<br>4.015
	Comparator	Exponential	Rate	0.05777	Log-logistic	Shape<br>Scale	2.139<br>2.351
1st-line treatment of advanced renal cell carcinoma	Intervention	Log-logistic	Shape<br>Scale	1.382<br>50.964	Log-normal	Meanlog<br>Sdlog	2.7714<br>1.2993
	Comparator	Log-normal	Meanlog<br>Sdlog	3.7437<br>1.5704	Log-normal	Meanlog<br>Sdlog	2.7714<br>1.2993

Abbreviations: NSCLC, non-small cell lung-cancer; TNBC, triple-negative breast cancer; RCC, renal cell carcinoma; HNSCC, head and neck squamous cell carcinoma; TPS, tumor proportion score

Supplementary Table S2. Key input data for the cost-effectiveness model

Indication	Health states	Cost, $US			Utility	
		Category	Intervention	Comparator	Intervention	Comparator
**Atezolizumab**						
2nd-line NSCLC (reimbursed indication)	PF	Treatment cost	2,970	820	0.74	0.74
		Dispensing cost	35	35		
		Monitoring and outpatient visit cost	233	233		
		Adverse event cost	23	441		
	PD	Subsequent treatment and monitoring cost	1,232	2,037	0.59	0.59
	Death	End-of-life cost	2,894	2,894	-	-
1st-line metastatic NSCLC	PF	Treatment cost (1~6 cycle)	7,279	4,310	0.71	0.71
		Treatment cost (after 7 cycle)	6,588	3,619		
		Dispensing cost (1~6 cycle)	57	57		
		Dispensing cost (after 7 cycle)	55	55		
		Monitoring and outpatient visit cost	239	239		
		Adverse event cost	717	534		
	PD	Subsequent treatment and monitoring cost	959	2,192	0.67	0.67
	Death	End-of-life cost	2,847	2,847	-	-
1st-line locally advanced or metastatic TNBC	PF	Treatment cost	4,658	1,540	0.715	0.715
		Dispensing cost	83	55		
		Monitoring and outpatient visit cost	418	557		
		Adverse event cost	175	150		
	PD	Subsequent treatment and monitoring cost	1,453	1,783	0.443	0.443
	Death	End-of-life cost	2,907	2,907	-	-
**Nivolumab**						
2nd-line NSCLC (reimbursed indication)	PF	Treatment cost	4,845	933	0.74	0.74
		Dispensing cost	50	34		
		Monitoring and outpatient visit cost	266	214		
		Adverse event cost	125	843		
	PD	Subsequent treatment and monitoring cost	1,129	1,548	0.59	0.59
	Death	End-of-life cost	2,978	2,978	-	-
2nd-line advanced RCC	PF	Treatment cost	4,819	1,325	0.895	0.895
		Dispensing cost	83	8		
		Monitoring and outpatient visit cost	311	260		

		Adverse event cost	9	45		
	PD	Subsequent treatment and monitoring cost	2,293	3,340	0.817	0.817
	Death	End-of-life cost	2,,941	2,941	-	-
2nd-line recurrent or metastatic HNSCC	PF	Treatment cost	4,819	3,707	0.805	0.77
		Dispensing cost	83	57		
		Monitoring and outpatient visit cost	185	189		
		Adverse event cost	6	27		
	PD	Subsequent treatment and monitoring cost	911	905	0.746	0.676
	Death	End-of-life cost	2,506	2,506	-	-
**Pembrolizumab**						
2nd-line NSCLC (reimbursed indication)	PF	Treatment cost	7,364	933	0.74	0.74
		Dispensing cost	34	34		
		Monitoring and outpatient visit cost	214	214		
		Adverse event cost	0	157		
	PD	Subsequent treatment and monitoring cost	686	1,279	0.59	0.59
	Death	End-of-life cost	2,978	2,978	-	-
1st-line treatment of metastatic non-squamous NSCLC	PF	Treatment cost	8,278	953	0.74	0.74
		Dispensing cost	57	57		
		Monitoring and outpatient visit cost	239	239		
		Adverse event cost	552	455		
	PD	Subsequent treatment and monitoring cost	939	2,766	0.67	0.67
	Death	End-of-life cost	2,847	2,847	-	-
1st-line treatment of metastatic squamous NSCLC	PF	Treatment cost	8,016	691	0.74	0.74
		Dispensing cost	57	57		
		Monitoring and outpatient visit cost	239	239		
		Adverse event cost	531	571		
	PD	Subsequent treatment and monitoring cost	618	948	0.67	0.67
	Death	End-of-life cost	2,847	2,847	-	-
1st-line metastatic NSCLC (≥50% TPS)	PF	Treatment cost	7,325	871	0.778	0.778
		Dispensing cost	57	57		
		Monitoring and outpatient visit cost	239	239		
		Adverse event cost	7	423		
	PD	Subsequent treatment and monitoring cost	231	353	0.641	0.641
	Death	End-of-life cost	2,847	2,847	-	-
1st-line treatment of	PF	Treatment cost	7,325	3,723	0.805	0.77

metastatic or unresectable recurrent HNSCC as monotherapy		(1~6 cycle)				
		Treatment cost (after 7 cycle)	7,325	3,661		
		Dispensing cost	55	55		
		Monitoring and outpatient visit cost	188	188		
		Adverse event cost	242	931		
	PD	Subsequent treatment and monitoring cost	1,481	1,778	0.746	0.676
	Death	End-of-life cost	2,506	2,506	-	-
1st-line treatment of metastatic or unresectable recurrent HNSCC as combination therapy	PF	Treatment cost (1~6 cycle)	7,440	3,723	0.805	0.77
		Treatment cost (after 7 cycle)	7,325	3,661		
		Dispensing cost	55	57		
		Monitoring and outpatient visit cost	188	188		
		Adverse event cost	1,074	981		
	PD	Subsequent treatment and monitoring cost	1,234	1,779	0.746	0.676
	Death	End-of-life cost	2,506	2,506	-	-
2nd-line recurrent or metastatic HNSCC (≥ 50% TPS)	PF	Treatment cost	6,790	3,707	0.805	0.77
		Dispensing cost	55	57		
		Monitoring and outpatient visit cost	185	188		
		Adverse event cost	8	101		
	PD	Subsequent treatment and monitoring cost	774	1,169	0.746	0.676
	Death	End-of-life cost	2,506	2,506	-	-
1st-line treatment of advanced renal cell carcinoma	PF	Treatment cost	9,275	2,815	0.75	0.75
		Dispensing cost	61	8		
		Monitoring and outpatient visit cost	253	231		
		Adverse event cost	160	111		
	PD	Subsequent treatment and monitoring cost	1,630	3,860	0.66	0.66
	Death	End-of-life cost	2,941	2,941	-	-

Abbreviations: PF, progression-free; PD, progressive disease; NSCLC, non-small cell lung-cancer; TNBC, triple-negative breast cancer; RCC, renal cell carcinoma; HNSCC, head and neck squamous cell carcinoma; TPS, tumor proportion score

Supplementary Table S3. Key input data for budget impact analysis

Non-reimbursed indication	The expected number of treated patients (the expected market share)			Annual cost, $US			
	The first year after expansion	The second year after expansion	The third year after expansion	Value-based pricng			4.75% price reduction
				Scenario 1: IBP	Scenario 2: IBP with refund	Scenario 3: WAP	
**Atezolizumab**							
1st-line metastatic NSCLC	1,977 (30%)	2,727 (40%)	3,526 (50%)	46,509,548	46,509,548	57,785,952	65,460,619
1st-line locally advanced or metastatic TNBC	181 (30%)	257 (40%)	341 (50%)	24,612,402	24,612,402	31,673,598	38,783,951
**Nivolumab**							
2nd-line advanced RCC	82 (30%)	114 (40%)	150 (50%)	34,661,572	24,837,802	30,639,859	23,678,067
2nd-line recurrent or metastatic HNSCC	297 (30%)	409 (40%)	527 (50%)	44,835,494	13,798,779	17,022,144	13,154,482
**Pembrolizumab**							
1st-line treatment of metastatic non-squamous NSCLC	1,384 (30%)	1,909 (40%)	2,468 (50%)	68,497,185	68,497,185	86,506,186	85,440,250
1st-line treatment of metastatic squamous NSCLC	824 (30%)	1,136 (40%)	1,469 (50%)	59,759,917	59,759,917	74,890,183	73,936,452
1st-line metastatic NSCLC (≥50% TPS)	655 (30%)	904 (40%)	1,169 (50%)	64,324,818	64,324,818	76,313,099	75,247,163
1st-line treatment of metastatic or unresectable recurrent HNSCC as monotherapy	506 (30%)	695 (40%)	896 (50%)	49,136,322	29,087,851	28,109,265	27,716,552
1st-line treatment of metastatic or unresectable recurrent HNSCC as combination therapy	506 (30%)	695 (40%)	896 (50%)	58,814,184	47,481,924	46,223,742	45,718,825
2nd-line recurrent or metastatic HNSCC (≥50% TPS)	155 (30%)	213 (40%)	274 (50%)	59,439,937	37,398,665	36,140,483	35,635,566
1st-line treatment of advanced renal cell carcinoma	248 (30%)	347 (40%)	455 (50%)	111,220,275	111,220,275	158,607,287	156,868,130

Abbreviations: IBP, indication value-based pricing; WAP, weighted average pricing; NSCLC, non-small cell lung-cancer; TNBC, triple-negative breast cancer; RCC, renal cell carcinoma; HNSCC, head and neck squamous cell carcinoma; TPS, tumor proportion score

Supplementary Table S4. Comparator and randomized clinical trial for each indication of immunotherapy

Indication	Intervention	Comparator	Randomized clinical trial
**Atezolizumab**			
2nd-line NSCLC (reimbursed indication)	Atezolizumab	Docetaxel	OAK study
1st-line metastatic NSCLC	Atezolizumab +bevacizumab +paclitaxel +carboplatin	Placebo +bevacizumab +paclitaxel +carboplatin	IMpower150
1st-line locally advanced or metastatic TNBC	Atezolizumab +nab-paclitaxel	Placebo +nab-paclitaxel	IMpassion130
**Nivolumab**			
2nd-line NSCLC (reimbursed indication)	Nivolumab	Docetaxel	CheckMate 017 +CheckMate 057
2nd-line advanced RCC	Nivolumab	Everolimus	CheckMate 025
2nd-line recurrent or metastatic HNSCC	Nivolumab	Docetaxel or Methtrexate or Cetuximab	CheckMate 141
**Pembrolizumab**			
2nd-line NSCLC (reimbursed indication)	Pembrolizumab	Docetaxel	KEYNOTE-010
1st-line treatment of metastatic non-squamous NSCLC	Pembrolizumab +platinum +pemetrexed	Placebo +platinum +pemetrexed	KEYNOTE-189
1st-line treatment of metastatic squamous NSCLC	Pembrolizumab +carboplatin +(nab)paclitaxel	Placebo +carboplatin +(nab)paclitaxel	KEYNOTE-407
1st-line metastatic NSCLC (≥50% TPS)	Pembrolizumab	Platinum based chemotherapy	KEYNOTE-024
1st-line treatment of metastatic or unresectable recurrent HNSCC as monotherapy	Pembrolizumab	Cetuximab +platinum +5-fluorouracil	KEYNOTE-048
1st-line treatment of metastatic or unresectable recurrent HNSCC as combination therapy	Pembrolizumab +platinum +5-fluorouracil	Cetuximab +platinum +5-fluorouracil	KEYNOTE-048
2nd-line recurrent or metastatic HNSCC (≥50% TPS)	Pembrolizumab	Docetaxel or Methtrexate or Cetuximab	KEYNOTE-040
1st-line treatment of advanced renal cell carcinoma	Pembrolizumab +axitinib	Sunitinib	KEYNOTE-426

Abbreviations: NSCLC, non-small cell lung-cancer; TNBC, triple-negative breast cancer; RCC, renal cell carcinoma; HNSCC, head and neck squamous cell carcinoma; TPS, tumor proportion score
